# Fitness of Crop-Wild Hybrid Sunflower under Competitive Conditions: Implications for Crop-to-Wild Introgression

**DOI:** 10.1371/journal.pone.0109001

**Published:** 2014-10-08

**Authors:** Kristin L. Mercer, D. Jason Emry, Allison A. Snow, Matthew A. Kost, Brian A. Pace, Helen M. Alexander

**Affiliations:** 1 Ohio State University, Department of Horticulture and Crop Science, Columbus, Ohio, United States of America; 2 University of Kansas, Department of Ecology and Evolutionary Biology, Lawrence, Kansas, United States of America; 3 Washburn University, Topeka, Kansas, United States of America; 4 Ohio State University, Department of Evolution, Ecology, and Organismal Biology, Columbus, Ohio, United States of America; University of Massachusetts, United States of America

## Abstract

Understanding the likelihood and extent of introgression of novel alleles in hybrid zones requires comparison of lifetime fitness of parents and hybrid progeny. However, fitness differences among cross types can vary depending on biotic conditions, thereby influencing introgression patterns. Based on past work, we predicted that increased competition would enhance introgression between cultivated and wild sunflower (*Helianthus annuus*) by reducing fitness advantages of wild plants. To test this prediction, we established a factorial field experiment in Kansas, USA where we monitored the fitness of four cross types (Wild, F_1_, F_2_, and BC_w_ hybrids) under different levels of interspecific and intraspecific competition. Intraspecific manipulations consisted both of density of competitors and of frequency of crop-wild hybrids. We recorded emergence of overwintered seeds, survival to reproduction, and numbers of seeds produced per reproductive plant. We also calculated two compound fitness measures: seeds produced per emerged seedling and seeds produced per planted seed. Cross type and intraspecific competition affected emergence and survival to reproduction, respectively. Further, cross type interacted with competitive treatments to influence all other fitness traits. More intense competition treatments, especially related to density of intraspecific competitors, repeatedly reduced the fitness advantage of wild plants when considering seeds produced per reproductive plant and per emerged seedling, and F_2_ plants often became indistinguishable from the wilds. Wild fitness remained superior when seedling emergence was also considered as part of fitness, but the fitness of F_2_ hybrids relative to wild plants more than quadrupled with the addition of interspecific competitors and high densities of intraspecific competitors. Meanwhile, contrary to prediction, lower hybrid frequency reduced wild fitness advantage. These results emphasize the importance of taking a full life cycle perspective. Additionally, due to effects of exogenous selection, a given hybrid generation may be especially well-suited to hastening introgression under particular environmental conditions.

## Introduction

Hybridization among differentiated plant taxa can introduce novel variation on which selection can then act (e.g., [1]). Thus, in any hybrid zone, questions arise about how hybridization and introgression (i.e., stable incorporation) may affect the evolutionary trajectories of the parent taxa and their hybrid progeny. Will there be introgression of introduced alleles into a given population or species? Further, which conditions promote or retard introgression? Addressing these questions requires an ecological genetics perspective, and much can be learned by measuring fitness components in common garden field experiments. Selection within hybrid zones can be environmentally independent and/or dependent, referred to as endogenous and exogenous respectively (reviewed in [2]). With endogenous selection, hybrid generations may have consistent, inherent fitness advantages or disadvantages relative to their parents and each other, i.e., due to the expression of genetic incompatibilities (e.g., [3]). By contrast, exogenous selection infers that selection operating on hybrids in hybrid zones may differ based on the environmental conditions. Such a situation implies the existence of genotype-by-environment (G×E) interactions, i.e., fitness differences among hybrids and their parents are influenced by local conditions (e.g., [4]). This environmental-dependence of the relative fitness of various hybrid generations can be crucial to evolutionary dynamics in hybrid zones [2], [5].

In this study, we explored the effect of the biotic environment on hybrid and wild parental fitness and specifically how inter- and intraspecific competition can affect rates of introgression. Interspecific competitors can differentially influence plant fitness and, ultimately, a genotype’s presence/dominance in that community [6]–[8]. Population density can be a major driver of the outcome of competition between species or among individuals within a species [9], [10] thereby influencing plant fitness. With regard to intraspecific competition, in addition to density, the genetic composition of intraspecific competitors can influence plant fitness. In hybrid zones, the relative frequencies of hybrids *vs.* non-hybrid genotypes are expected to vary depending on the distances between hybridizing individuals, the relative sizes of each population, pollinator behavior (if relevant), and other ecological factors [11], [12]. When the frequency of particular genotypes or cross types within a population affects fitness, frequency-dependent selection also has the capacity to influence introgression dynamics [13]–[15]. Knowledge of the differential ability of hybrids and parental types to complete their entire life cycle within the context of a range of realistic competitive environments should allow us to better predict the conditions under which we may or may not see introgression. However, studies that provide these data are rare, as described in [36].

A common and well-studied case of hybridization between differentiated populations is gene flow between crops and their wild relatives [11]. The advent of genetically modified crops raised concerns about the potential for introgression of novel alleles into wild populations [16], [17] possibly increasing invasiveness or altering the wild population’s genetic structure (see [11] for review). The possibility of introgression of crop alleles, and the rate at which it occurs, depends on the fitness of various hybrid generations relative to their wild counterpart [18]. Yet the relative fitness of wild and crop-wild hybrid generations can be strongly influenced by the environment in which they are compared (e.g., [19]). Biotic and abiotic factors that can affect hybrid fitness include pathogen or herbivore species [20]–[23], unspecified differences between locations [24], [25], and competitive conditions [19], [26], [27], among others. Many fitness studies are performed in highly controlled conditions (e.g., in a greenhouse: [20]) or in field conditions mimicking farm fields (e.g., [19]) even though crop-wild hybrid zones often extend into non-cultivated areas. To clarify the context dependence of crop allele introgression on the unmanaged landscape, we need field studies that rigorously address how fitness of multiple hybrid generations is influenced by a range of relevant biotic conditions [28].

Our current research focuses on *Helianthus annuus* (common sunflower), which exhibits a particularly high gene flow rate in the USA, with as many as 66% of cultivated fields surveyed overlapping in flowering time with an adjacent, conspecific wild population [29]. Up to 25% of seeds produced in wild sunflower populations alongside crop fields have been shown to be crop-wild hybrids; such hybrids have also been found in populations up to 1 km away from crop fields [30]. Moreover, crop alleles can remain in wild populations for >5 years and crop-to-wild introgression can be relatively common [31], [32]. In past studies, we found that F_1_ crop-wild hybrid sunflowers produced fewer seeds per plant relative to wild sunflowers, but this disadvantage of hybrids diminished when compared under more competitive conditions [19], [33], indicating that rates of introgression might increase accordingly under such conditions. Increased competition reduced branching in wild genotypes, while the faster seedling growth in the hybrids may have increased their performance under a denser canopy [34]. However, this past work only considered F_1_ hybrids, and thus did not include many of the hybrid generations found in hybridizing wild sunflower populations. Further, it did not assess fitness throughout the life cycle under a range of natural competitive environments.

Here we report findings from a large, manipulative competition experiment in Kansas, USA. We measured the survival and fecundity of wild and three crop-wild hybrid sunflower cross types grown for their entire life cycle under a range of competitive conditions in the field. First, we hypothesized that the presence of interspecific competitors and increased density of intraspecific competition would reduce fitness advantages of the wild plants relative to the hybrids [19]. This was confirmed, although wild plants still retained superior fitness over hybrids, especially once we accounted for their better overwintering seed survival and seedling emergence. Second, we predicted that a higher frequency of the faster growing F_1_ seedlings should lead to a more competitive environment and a reduction in the wild advantage over hybrids. This prediction was not upheld and we found that the effects of frequency were minor relative to the other treatments. We conclude that knowledge of the differential ability of hybrids and wild types to survive and reproduce across this range of competitive environments should allow us to better predict the biotic conditions that affect introgression.

## Methods

### Seed sources

Seed sources and crossing design for the hybrid generations are explained in Weiss et al. [35] and Alexander et al. [36]. In brief, we collected achenes (hereafter, seeds) from common sunflower (*Helianthus annuus*) populations in and around Lawrence, Kansas, in the fall of 2006 from five habitats where common sunflower is often found: roadside, construction zone, agricultural field, abandoned field, and wetland. Crop sunflowers are uncommon in northeastern Kansas. By collecting from these environments, we attempted to include all possible adaptive diversity from the local gene pool. Seeds from these original populations were pooled and sown for use in hand-pollinations in Columbus, OH, in 2007 to produce an F_1_ hybrid cross type between the wild and crop sunflower. A crop sunflower inbred line known as HA 89 was used as the pollen parent for F_1_ crosses. Crop-wild F_1_ hybrid progeny produced on 20 wild maternal parents in 2007 were then used in 2009, along with the original wild seeds and HA 89, to produce four cross types: a new set of wild (wild×wild) and F_1_ hybrid (wild×crop) progeny, as well as F_2_ hybrid (F_1_×F_1_) and BC_w_ hybrid (wild×F_1_) generations. (Maternal parent is noted first in the parenthetical crosses). As discussed more fully in Alexander et al. [36], F_1_ plants from these populations appeared to be self-incompatible (KLM, personal observation). Wild, F_1_, and BC_w_ cross types were produced on the same 18 wild maternal parents, while F_2_ seeds were produced on 18 F_1_ maternal parents. Wild crosses on a given maternal parent were sired by approximately five wild pollen parents, while F_1_ crosses were sired by up to five pollen parents of the genetically uniform crop parent. BC_W_ and F_2_ crosses were sired with pollen from two F_1_ pollen parents or with bulked F_1_ pollen from multiple pollen parents. Seeds from up to five inflorescences (hereafter, heads) were used to make up a given maternal family. With this procedure, all seeds for the four cross types were of similar age and were produced on bagged inflorescences of field-grown plants.

### Establishment of the field experiment

Seeds of these four sunflower cross types were then used to establish a large field experiment in the fall of 2009 at the University of Kansas Field Station (Jefferson County, Kansas, USA). We planted seeds in the fall because natural seed dispersal occurs from October - December; this planting time allowed for fitness to include the ability of seeds to overwinter. Overall, seeds used in this study represented three categories: focal, matrix, and buffer seeds. The focal seeds represented all four cross types and we followed their fate from emergence through seed production. Matrix seeds consisted of mixtures of wild and F_1_ crop-wild hybrid seeds and were used to create the different intraspecific competition treatments (density and frequency of hybrids). Buffer seeds were wild seeds collected from our crossing blocks that were spread in the outer 15 cm of each plot to reduce edge effects.

In overview, the experiment consisted of six blocks that were established in an old field environment dominated by brome grass (*Bromus* spp.) in November 2009. (Blocks had been rototilled in the spring of 2009 to allow for emergence of weeds and to confirm that wild sunflower was not in the seed bank). Within each block, we established two 14.3 m×1.35 m strips with a wide aisle between them, which together contained a total of twelve 1.35 m×1.8 m plots. As described in detail below, these 12 plots consisted of factorial combinations of levels of the three competitive factors. We refer to these as environmental treatments. Within each plot, we followed the fate of 72 focal seeds (four cross types×18 families per cross type); these will be referred to as genetic treatments. From a statistical perspective, this is a split plot design with randomized locations of the environmental treatments ( = main plots) within each block and randomized locations of genetic treatments within each main plot ( = subplots).

The 12 environmental treatments were factorial combinations of the various levels of an interspecific competition factor and two intraspecific competition factors (density of seed rain (hereafter, seed density) and crop-wild hybrid frequency). We chose to manipulate competition around the focal plants because wild sunflower populations establish within various vegetation contexts–from monospecific stands to highly diverse communities (H.M.A, Pers. Obs.) and from low to high densities [37], [38]. To create two levels of interspecific competition, we either weeded all plants other than matrix sunflower (often ragweed) or allowed these species and sunflowers to coexist. We created three levels of intraspecific seed density (100, 255, and 495 seeds m^−2^) by altering the amount of matrix seeds. We manipulated a second intraspecific competitive factor, the frequency of hybrids, by altering the frequency of F_1_ seeds in the matrix seeds to mimic wild populations experiencing substantial gene flow from cultivated sunflower fields. We chose two levels, 15% and 40%. Arias and Rieseberg [30] found that crop-wild hybrid sunflower seeds can be produced at a frequency of as high as 0.60 on plants just three meters from a crop field (average of 0.27), while that frequency drops to 0.10 by 300 m and to closer to 0.01 by 1000 m. See Text S1 for analyses to confirm that our manipulations were successful in altering these three factors.

Into each plot experiencing a given set of environmental treatments, we planted our genetic treatments (72 focal seeds = 4 cross types×18 families per cross type). Due to incomplete emergence, families could not be included as a factor in our analyses of fitness and will not be discussed further. Focal seeds were affixed to labeled plastic cocktail stirrers (hereafter, swizzle sticks) with Gorilla Glue (Gorilla Glue Company, Cincinnati, Ohio) to allow us to maintain the identity of emerging focal seedlings in the following spring, as in Mercer et al. [39]. We planted focal seeds just below the soil surface at 10 cm spacing. We then scattered matrix seeds over the whole plot while protecting each focal seed with a small cup to maintain a small, cleared zone around it. After removing the cups, we covered the area with a 1 cm layer of sieved field soil. Focal seeds were planted in the center portion of each plot and excluded from a 15 cm buffer zone to reduce edge effects on fitness measures. We lowered sample sizes of hybrid cross types in the low density, low hybrid frequency plots to maintain the correct hybrid frequency. In total, we planted 4824 focal seeds in November 2009. The focal seeds that successfully overwintered and emerged in the spring were marked as focal plants and were observed for the rest of the season. See Table S1 for the sample sizes of focal seeds that emerged from each treatment combination.

### Data collection

We focused on components of lifetime fitness of focal seeds, namely emergence, survival to reproduction, number of mature heads per plant, and seeds produced per head. Focal seedlings began to emerge March 22, 2010, and emergence was monitored every two to three days until May 27, when emergence became rare (i.e., only one seedling had emerged in eight days). At the end of the season, we counted the number of heads per plant. Sunflower plants typically have only one primary head (derived from their apical meristem) and can have numerous secondary heads (here defined as heads that are produced on ends of branches or on branches off branches). However, damage to the apical meristem can lead to loss of the primary head; such plants have increased branching and thus more secondary heads. We categorized developing primary and secondary heads as high or low quality. Low quality heads were those covered in larval frass or either too hard or too soft in the bud stage–all harbingers of poor or absent seed development (see Alexander et al. [36] for further discussion). Heads that did not have time to mature before killing frosts were not counted.

To estimate seed production, we collected the primary and first secondary head produced on each plant and counted their seeds by hand. All other secondary heads were randomly subsampled in such a way that any given head had an 80% chance of being selected for collection and counting. When seeds were counted in a given head, we noted whether each seed was in good condition or whether it had a hole in the pointed or blunt end or a bite removed from the pointed end. Holes towards the seed’s pointed end were likely made by *Isophrictis similiella* (Lepidoptera); holes towards the seed’s blunt end were likely caused by weevils (*Smicronyx fulvus* and *S. sordidus*, Coleoptera); and the insects that made the larger bites in the seeds are unknown [40] (D. Pilson, personal communication). Only good quality seed were included here in fitness estimates. Low quality heads covered in larval frass (noted above) had been infested with *Homeosoma electellum* larva, which resulted in total seed loss in most affected heads, thereby reducing overall fitness. The use of bridal veil to cover maturing seed heads may have deterred some seed predators, including other insects and birds, although we do not expect this to be a major factor in estimating seed production. The apical meristems of some plants at our site were attacked by disease, stem borers, or gall producing insects, resulting in unusually high levels of branching and head production. We collected data on the presence of this meristem damage during the season in order to be able to take it into account in our analysis.

### Data analysis

We used Glimmix in SAS (version 9.3) to run restricted maximum likelihood ANOVAs to test the effects of our environmental and genetic treatments on our fitness response variables. Given the split-plot design, we tested for the effects of our main plot factors – seed density, hybrid frequency, and interspecific competition (and their interactions) – using the interaction of the block, seed density, hybrid frequency, and interspecific competition factors as an error term. Analysis of our genetic treatment was restricted to cross type since family was poorly replicated, as noted above; thus, cross type was applied at the subplot level and its effect and all of the interactions between cross type and our three main plot factors were tested with the pooled error term composed of the interaction of the block, seed density, hybrid frequency, interspecific competition, and cross type factors. Blocks were considered random, as were any interactions with block.

Fitness estimates depended on a) probabilities of reaching life cycle stages (such as becoming reproductive) and b) seed production per reproductive plants. For the former, we first analyzed the probability that focal seeds planted in the fall emerged and produced a mature head (i.e., survived to reproduce). Least squares means of these probabilities were predicted using the binomial distribution option in Proc Glimmix accounting for all treatment effects (as above). Probabilities for individuals surviving to reproduce were conditional on emergence. (See Table S1 for sample sizes for number of focal seeds that emerged and survived to reproduce). For seed produced per reproductive plant, we used estimated numbers of seeds per head and known counts of numbers of heads. Estimates of number of seeds per head for a particular plant were based on knowledge of whether the head was primary or secondary (and high or low quality) and the cross type and environmental treatments of the plant (Text S2, Table S2).

We estimated seed production per reproductive plant as:

(presence of high quality primary head×num. viable seeds per high quality primary head) + (presence of low quality primary head×num. viable seeds per low quality primary head) +
(num. high quality secondary heads×num. viable seeds per high quality secondary head) +
(num. low quality secondary heads×num. viable seeds per low quality secondary head).

While this estimated seed per reproductive plant, we ultimately produced average estimates for each cross type in each plot for subsequent analyses. Similarly, we created two increasingly integrative fitness measures. First, also by cross type and plot, we estimated the number of seeds produced per emerged seedling. This was defined as the probability of survival to reproduction×number of seeds produced per reproductive plant. Second, we defined the number of seeds produced per planted seed as the probability of emerging×probability of survival to reproduction×number of seeds produced per reproductive plant. These analyses of seed numbers included a binary variable for meristem damage to account for effects on seeds via enhanced head production.

Subsequent calculations of the fitness of a given hybrid cross type, *i*, relative to the wild (relative fitness, w*_i_*) were simply calculations of wild fitness/hybrid fitness in the same set of treatment combinations and employed back-transformed least squares means from the analyses above. Calculations of the % change in w*_i_* as interspecific competition was added was calculated as (w*_i_* with interspecific competition – w*_i_* without interspecific competition)/w*_i_* without interspecific competition for each level of intraspecific density. Similarly, the % change in w*_i_* as density of intraspecific competitors increases = (w*_i_* under high density – w*_i_* under low density)/w*_i_* under low density for with and without interspecific competition. Finally, the % change in relative fitness going from low density, without interspecific competitors to high density, with interspecific competitors = (w*_i_* high density, with interspecific competition – w*_i_* low density, without interspecific competition)/w*_i_* low density, without interspecific competition.

## Results

### Effects of competitive treatments and cross type identity on emergence and survival to reproduction

Competitive environment did not differentially affect the emergence of cross types and only cross type itself had a significant effect on seedling emergence in the spring (Table 1). Wild seed emerged at the highest proportion (0.68, s.e. = 0.017), followed by BC_w_ (0.61, s.e. = 0.019), F_1_ (0.50, s.e. = 0.019), and F_2_ seeds (0.40, s.e. = 0.018) (LS means; all significantly different with a Tukey-Kramer test). By contrast, only density of intraspecific competitors affected the probability of survival to reproduction once a seedling had emerged (Table 1). Plants in low and medium density plots had high probabilities of reproducing (low density: 0.92 (s.e. = 0.013); medium density: 0.87 (s.e. = 0.015)), while the probability was 0.74 (s.e. = 0.023) for high density plots (low and medium significantly different from high, Tukey-Kramer test).

**Table 1:** ANOVA on fitness measures for crop-wild hybrid sunflowers grown under competitive conditions in Kansas, USA.

			Prob. Emerged^1^		Prob. Surv. Reprod.^1^		Seeds per Repr. Plt^2^		Seeds per Emgd Sdling^2^		Seeds per Seed Plted^2^	
Source	ndf^3^	ddf^3^	F	P-value	F	P-value	F	P-value	F	P-value	F	P-value
Frequency of Hybrids	1	55	0.23	0.6371	0.14	0.7102	6.19	**0.0159**	7.57	**0.008**	5.46	**0.0231**
Interspec Competitors	1	55	0	0.9707	0.74	0.3947	61.06	**<.0001**	50.19	**<.0001**	36.72	**<.0001**
Density of Seeding	2	55	0.06	0.9456	33.41	**<.0001**	292.8	**<.0001**	315.6	**<.0001**	215.53	**<.0001**
Freq*Interspec	1	55	0.04	0.8514	0.26	0.6138	0.52	0.4742	0.23	0.63	0	0.9872
Freq*Dens	2	55	1.07	0.3499	0.08	0.9254	1.81	0.1736	1.85	0.167	2.56	0.0864
Interspec*Dens	2	55	0.05	0.9498	0.18	0.8333	2.9	0.0636	3.14	0.0513	3.14	0.0511
Freq*Interspec*Dens	2	55	0.47	0.6251	0.92	0.4063	0.08	0.9229	0.01	0.9907	0.36	0.6993
Cross Type	3	179–186	65.09	**<.0001**	1.45	0.2305	197.9	**<.0001**	159.5	**<.0001**	168.17	**<.0001**
Freq*Cross	3	179–186	0.24	0.8667	1.55	0.2036	1.66	0.1778	4.09	**0.0078**	2.81	**0.0412**
Interspec*Cross	3	179–186	1.18	0.3178	0.75	0.5227	1.06	0.3679	1.8	0.1492	1.88	0.1341
Dens*Cross	6	179–186	0.59	0.7406	1.48	0.1875	14.68	**<.0001**	13.39	**<.0001**	11.52	**<.0001**
Freq*Interspec*Cross	3	179–186	0.29	0.8338	0.68	0.5654	0.93	0.4292	0.39	0.7576	0.46	0.7125
Freq*Dens*Cross	6	179–186	0.59	0.7414	0.49	0.8174	0.69	0.6548	0.48	0.8259	0.63	0.7057
Interspec*Dens*Cross	6	179–186	0.31	0.9306	0.33	0.9185	2.35	**0.033**	2.19	**0.046**	1.4	0.2167
Freq*Interspec*Dens*Cross^4^	6	179–186	0.94	0.4668	–	–	0.12	0.9945	0.22	0.9687	0.55	0.7677
Meristem Damage	3	1941	–	–	–	–	3.95	**0.0485**	0.11	0.7377	2.53	0.1138

Notes: These analyses explore the effects of experimental treatments on the probability that focal seeds emerged and survived to reproduce, the number of seeds produced by reproductive plants, the number of seeds produced by emerged seedlings, and the number of seeds produced by each seed planted the previous fall (see text for details). P-values>0.05 are bolded to emphasize significance.

1 Binary life cycle data (probability of emergence and survival to reproduction) analyzed using residual pseudo-likelihood and the logit link function in SAS Glimmix.

2 Seeds per reproductive plant, Seeds per emerged seedling, and Seeds per planted seed were all natural log transformed (ln(y+1)).

3 ndf and ddf, numerator and denominator degrees of freedom for each trait, respectively.

4 This 4-way interaction was left out of the probability of survival to reproduction analysis because the model could not otherwise converge.

### Differential effects of competition on seed production of the cross types

Given our original hypotheses regarding how the fitness of crop-wild hybrids relative to their wild counterparts would increase under more competitive conditions, we were most interested in discerning how interactions between competitive factors and cross type influenced seed production (i.e., G×E interactions). If found, the presence of such higher order interactions makes discussion of main effects irrelevant (Table 1).

**Figure 1:**
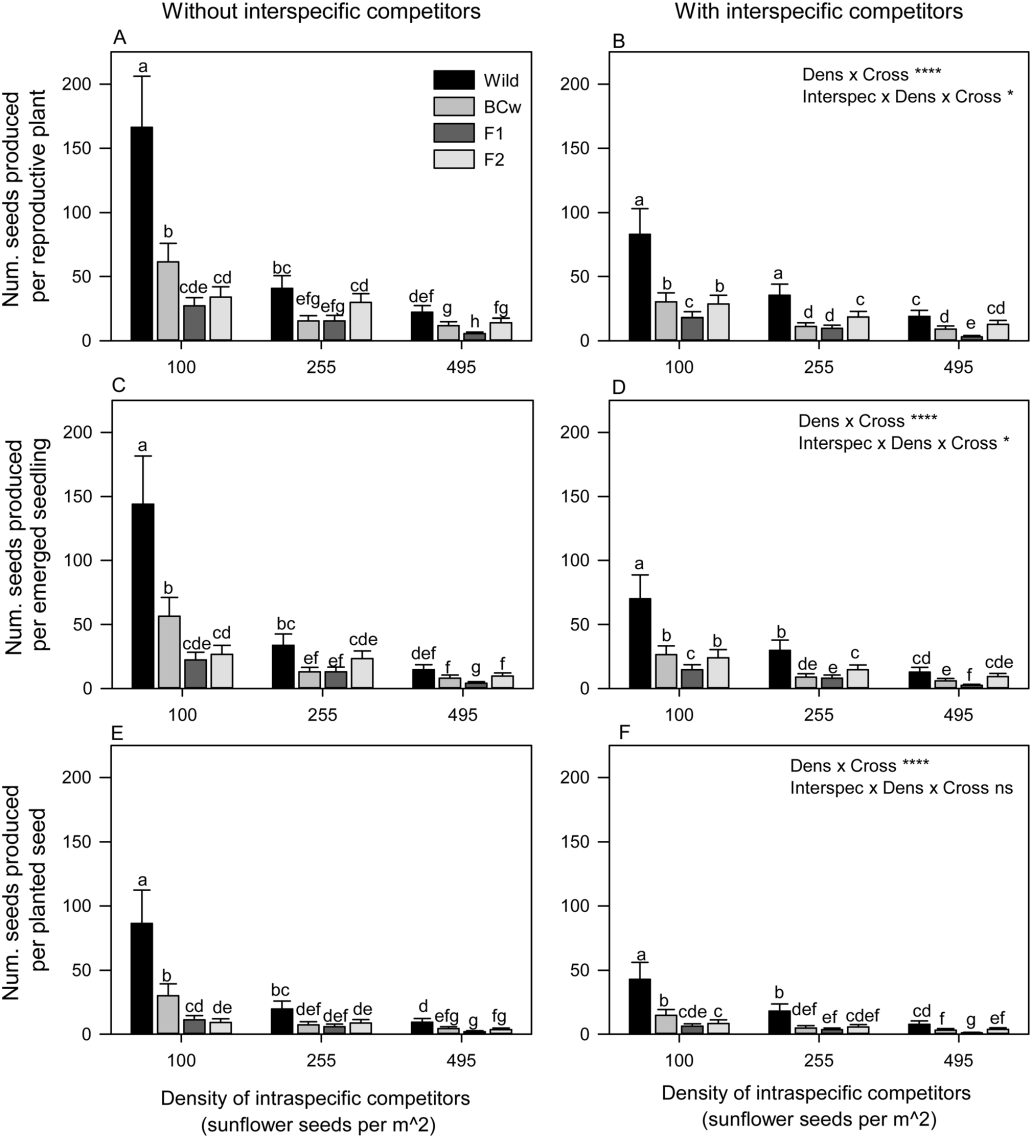
Combined effects of interspecific competitors, density of intraspecific competitors, and crop-wild hybrid cross type on three fitness measures in sunflower. Three fitness measures integrate increasing proportions of the life cycle: number of seeds produced per reproductive plant (A, B), number of seeds produced per emerged seedling (C, D), and number of seed produced per planted seed (E, F). Values are back-transformed least squares means with 95% confidence intervals (only the larger upper portion of asymmetrical interval is shown). Values sharing the same letter within a panel are not significantly different based on Tukey-Kramer multiple comparison tests. ANOVA effects for reference correspond to Table 1. Dens = Density of intraspecific competitors; Cross = Cross type; Interspec = Interspecific competition; *P<0.05, ****P<0.0001, ns P≥0.05.

The interaction between cross type, the density of intraspecific competitors, and the presence of interspecific competitors had the greatest effect on changing the magnitude of fitness measures, which largely supported our expectations (Table 1, Figure 1). Under the least competitive conditions (low density, no interspecific competition), wild plants were far more fit than BC_w_, which were more fit than F_1_ or F_2_ cross types (Figure 1). However, fitness differences among cross types were much reduced in magnitude under the more competitive high density treatments or when interspecific competition was applied (Figure 1). (Fitness differences would have appeared eliminated had we only assayed heads per plant (Figure S1)). In fact, for the number of seeds produced per reproductive plant, the difference between the wild and F_2_ cross types was 22 times as great under low density, without interspecific competition (Figure 1A) as under high density, with interspecific competition (Figure 1B). Thus, F_2_ hybrids could not be distinguished from wild plants under medium density, without interspecific competition (Figure 1A) or at high densities, with interspecific competition (Figure 1B). Moreover, once survival to reproduction was also taken into account, there were even more cases where more competitive conditions eliminated the fitness differences between wilds and particular hybrid generations (in this case, both F_2_ and BC_w_ hybrids; Figure 1C, D, Table 1). However, when emergence of seeds in the spring was included in the compound fitness measure (i.e., for seed production per planted seed), none of the hybrids were equivalent to wild cross type, even under high density (Figure 1E, F). Still, the magnitude of difference between wild and F_2_ cross types remain 19 times greater under low density, without interspecific competition (Figure 1E) as under high density, with interspecific competition (Figure 1F). Therefore, for seed production per planted seed, the wild cross type ultimately maintained a slight fitness advantage, despite responding more negatively to the competitive conditions than certain hybrids.

Assessing this same G×E interaction using relative fitness values rather than absolute values can further illustrate selection pressures. Using the most complete fitness measure, numbers of seeds produced per planted seed, we calculated values of the fitness of each hybrid cross type relative to the wild under factorial combinations of interspecific competition (with and without) and under the highest and lowest densities of intraspecific competition. These relative fitness values ranged from 0.34–0.46 for BC_w_, 0.13–0.20 for F_1_, and 0.11–0.48 for F_2_ cross types (Table 2). None of these values is greater than one and as we compare treatment combinations (i.e., from low to high density, with interspecific competitors), we see no large rearrangements in ordering or fitness rankings. However, relative values do change in response to treatments. All of the lowest relative fitness values are found under low density and all the highest are found under high density, although F_1_ values remain generally low throughout. Increasing density, without interspecific competition, had the greatest positive effect on relative fitness for BC_w_ and F_1_ cross types, with changes of 32% and 56%, respectively (Table 2). By contrast, adding interspecific competition and increasing density increased relative fitness for F_2_ cross types by 353% (Table 2). On the low end, relative fitness values for BC_w_ and F_1_ cross types declined with interspecific competition under high density by 12 and 33%, respectively (Table 2). Under those same conditions, F_2_ relative fitness increased by 26%, which was by far the smallest change seen in F_2_ relative fitness with increased competition. As a caveat, without error terms on these estimates, it is hard to know which differ significantly from zero.

**Table 2:** Relative fitness and percent changes in relative fitness for crop-wild hybrid sunflowers grown under competitive conditions in Kansas, USA.

		Density of intraspecific competitors								
		Low (100 seeds per m^2^)			High (495 seeds per m^2^)			% Change as density increases		
		Cross type			Cross type			Cross type		
		BC_W_	F_1_	F_2_	BC_W_	F_1_	F_2_	BC_W_	F_1_	F_2_
InterspecificCompetition	Without	0.35	0.13	0.11	0.46	0.20	0.38	32%	56%	260%
	With	0.34	0.14	0.20	0.41	0.13	0.48	19%	−6%	146%
% Change as interspecificcompetition added		−2%	11%	84%	−12%	−33%	26%	16%^1^	5%^1^	353%^1^

Note: Relative fitness, change in relative fitness, and cross types are as defined in the text (Materials and Methods section).

1 These cells represent changes in relative fitness from low density, without interspecific competition to high density, with interspecific competition.

**Figure 2:**
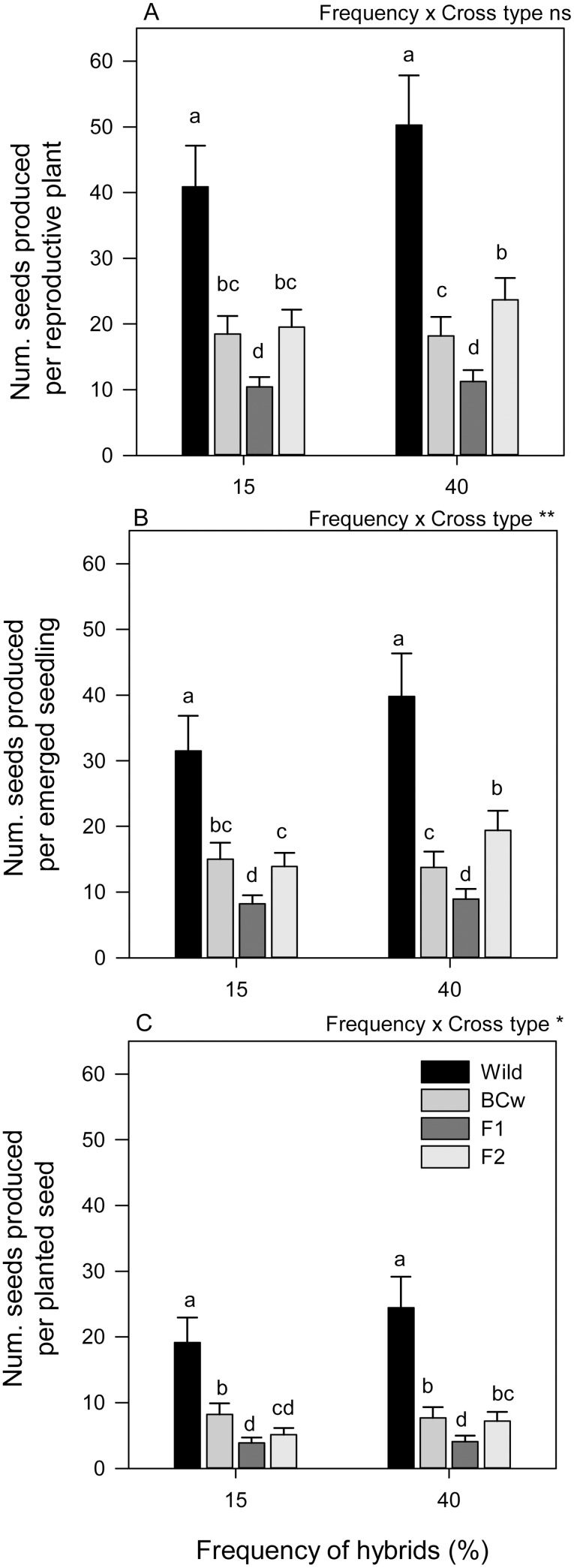
The combined effects of the frequency of crop-wild hybrids and cross type on three fitness measures in sunflower. Three fitness measures integrate increasing proportions of the life cycle: number of seeds produced per reproductive plant (A), number of seeds produced per emerged seedling (B), and number of seed produced per planted seed (B). Values are back-transformed least squares means with 95% confidence intervals (only upper portion of asymmetrical interval is shown). Values sharing the same letter within a panel are not significantly different based on Tukey-Kramer multiple comparison tests. ANOVA effects for reference correspond to Table 1. *P<0.05, **P<0.01, ns P≥0.05.

The frequency of hybrids also differentially affected the fitness of cross types (Table 1, Figure 2). For two cross types (F_1_ and BC_w_), seed production was equivalent across the treatments, and for two others (Wild and F_2_), seed production decreased (or trended to) as frequency of hybrids declined (Figure 2). Importantly, the magnitude of differences among cross types declined with decreases in hybrid frequency due to non-significant, but substantial reductions in wild seed production (Figure 2). Declines in hybrid frequency also tended to align the seed production of the F_2_ and BC_w_ cross types (Figure 2A, B). However, once emergence was accounted for, the F_2_ and BC_W_ cross types were equivalent no matter the hybrid frequency (Figure 2C). Ultimately, we did identify differences in the fitness of the hybrid cross types relative to the wild in seeds produced per planted seed as hybrid frequency declined: both the BC_W_ and F_1_ cross types increased in relative fitness (37% and 21%, respectively); the relative fitness of the F_2_ declined slightly by 9%. Regardless, changes in hybrid frequency did not have a strong enough effect to eliminate the wild fitness advantage over the various hybrids, no matter the measure.

## Discussion

### Effects of environmental treatments on hybrid vs. wild fitness

The likelihood of crop alleles introgressing into wild populations depends on the overall fitness of early-generation hybrids, plus the fitness effects of particular alleles and any linked loci [41]. We found evidence that fitness of crop-wild hybrids relative to wild sunflowers was not constant, but depended on the competitive context. We can thus begin to infer the ecological conditions consistent with higher rates of introgression and the cross types that are more likely to contribute to that introgression.

Specifically, we had predicted that competitive environments created with the presence of interspecific competitors, high intraspecific density, and higher hybrid frequency might enhance introgression by increasing the fitness of crop-wild hybrids relative to wild genotypes. Our data supported some of these predictions, but not all. We did find that higher density and the presence of interspecific competitors generally increased the fitness of hybrids relative to the wild, especially for the F_2_. High competition reduced the magnitude of differences between cross types, such that wild genotypes became indistinguishable from some hybrids (F_2_ and occasionally BC_W_) in terms of seeds per emerged seedling (Figure 1C, D). Yet using our most complete fitness measure, seeds per planted seed, hybrid and wild fitness was never equivalent (Figure 1E, F). Hybrid frequency also affected fitness, although low frequency of hybrids proved to be the set of conditions which reduced fitness differences between cross types (Fig. 2). This latter observation was contrary to our initial prediction, but previous work confirms that increasing hybrid frequency can reduce the relative fitness of hybrids [26] and that frequency-dependent responses can be quite profound, even causing reversals of rankings among hybrid cross types [27]. Thus, habitats with high intraspecific density, with the presence of interspecific competitors, or with low hybrid frequencies may ultimately increase the likelihood of crop allele introgression.

### Exogenous selection by intra- and interspecific competition

Our study is consistent with the phenomenon of exogenous selection acting within crop-wild hybrid zones. For some studies of exogenous selection, such as where there is local adaptation across an environmental cline [42], one might expect reversals of rankings of genetic classes across that cline, so relative fitness values would shift dramatically [2], [5]. However, for investigations of G×E interactions that could promote introgression under particular sets of conditions, reversals of fitness rankings may not be apparent or necessary. In our experiment, competition greatly reduced the magnitude of differences in fitness among cross types, but we did not see any radical shifts in their rankings. Wild plants maintained an actual, if not statistical, advantage over most hybrid classes across most components of fitness (i.e., from emergence through seeds per seed planted). Yet the relative fitness of all hybrids tended to increase with competition (Table 2). In other crop-wild hybrid systems, competition has also been shown to increase the relative fitness of hybrids, though not always ([26] and references therein). Our work adds to the few studies that have dissected the effects of different forms of competition on hybrid fitness (e.g., [26], [27]). To our knowledge, ours is the first study of its type that has followed plants from seed to seed under field conditions.

It is important to emphasize that, despite the lack of radical shifts in ranking, the effects of competition on hybrid fitness were not equivalent and relative fitness values did change across competition treatments. F_1_ and BC_W_ relative fitness values were mildly responsive to density; they both changed only an average of 25% as density increased (Table 2). By contrast, the exogenous selection affecting the F_2_ cross type was stronger. Increasing density of intraspecific competitors more than doubled or tripled the fitness of the F_2_ cross type relative to its wild counterpart and increasing density while also adding interspecific competitors quadrupled it (Table 2). This magnitude of change in relative fitness should be sufficient to alter evolutionary processes and enhance introgression of crop alleles under these biotic conditions. Thus, the F_2_ cross type will be much more likely to ferry crop alleles to the subsequent generation under competitive conditions than when competition is low.

### Factors contributing to competitive resilience in F_2_

While the BC_W_ cross type did best among the hybrids under lower competition, the F_2_ cross type equaled or surpassed it as competition increased (Figure 1). Several factors could account for improved competitive ability in F_2_ progeny. The first relates to differences between F_2_ progeny and their fellow hybrids with 50% crop contribution–the F_1_ cross type. By having different maternal parents (F_1_ seeds were produced on a wild maternal plant and F_2_ seeds were produced on an F_1_ maternal plant), maternal genetic effects could produce cross types differences due to any maternally inherited seed coverings, organelles, or organellar genomes [43]. The seeds and seedlings of the F_2_ cross type were larger than those of the F_1_; this could have enhanced competitive ability as seedling size was associated with a greater chance of surviving to flowering in this same experiment (MAK, personal observation). Others have seen similar fitness benefits of seed or seedling size under competitive conditions [44], [45]. It should be noted, however, that in a sunflower study with lower competition, maternal genetic effects in sunflower were not observed to affect fitness late in the life cycle [36]. Large seed size can also have negative implications in this species, such as higher herbivory [38].

Increased F_2_ competitive ability could also be the result of selection earlier in the season. Because F_2_ genomes constitute recombining wild and crop genomes, particular combinations of homozygous wild loci may lead to individuals that have wild phenotypes and surprisingly high fitness. Likewise, individuals that are crop-like at these loci could have experienced premature germination or overwintering mortality [36], as evidenced by the F_2_ cross type having the lowest emergence. Thus, strong selection during the overwintering phase may have selected for a more fit or competitive subgroup of the F_2_ progeny, if alleles on which selection was operating were physically linked to, or had pleiotropic effects on, traits with effects during later life stages.

### Life cycle stages and fitness estimates

It is rare for researchers to label individual seeds of known genetic background and follow their performance to the next generation while creating a realistic competitive environment. By taking this approach, we were able to include a seed’s overwintering survival and successful germination as components of fitness. Yet there still remain gaps in our understanding of seed-related fitness components. For instance, premature germination of hybrid seeds prior to the spring [35], [36] may be what reduced the most comprehensive fitness measure, seeds produced per planted seed, and kept wilds and hybrids from parity. On the other hand, the comprehensive estimates of F_2_ fitness may actually be conservative here because an adjacent study registered higher emergence for the F_2_ than for wild seedlings [36]. Thus, wild and hybrid equivalence might be possible for all fitness metrics under competitive conditions. Nevertheless, we could not account for seed dormancy in this study. Including dormancy would have likely further enhanced the fitness of cross types produced on the wild maternal parent–especially the wilds themselves. Ungerminated wild seeds are more likely to overwinter safely, remain dormant in the soil seed bank, and may emerge another year, while ungerminated hybrid seeds with more crop-like maternal parents and greater percentages of crop ancestry are more likely to die as seeds or prematurely germinate [46]. Clearly, accounting for early stages of the life cycle facilitates a better understanding of fitness and relative fitness differences among hybrid cross types. We are aware, however, that our results may depend on the unique abiotic and biotic conditions present in the year of our study.

Overall, our results showed that the fitness deficits experienced by crop-wild hybrids compared to wilds were diminished when various forms of inter- and intraspecific competition were applied, indicating the potential for exogenous selection within naturally occurring crop-wild hybrid zones. However, using more complete information from across the life cycle also clarified that early traits (seed overwintering ability, emergence, seedling size) can reduce hybrid fitness, but also play a role in enhancing the competitive ability of some cross types (e.g., F_2_). These early traits are all controlled to some extent by maternal genetic effects, yet the potential role of maternal genetic effects in altering rates of introgression does not appear to be well-studied, especially beyond the F_1_ generation (but see [47]). Further, maternal effects are notoriously difficult to account for in evolutionary processes more generally [48]. Crop-wild hybrid zones and other (animal or plant) hybrid zones where taxa are differentiated for traits controlled by maternal genetics may prove to be excellent laboratories for such study. In conclusion, full life cycle assessments of fitness differences among hybrid generations are useful for assessments of opportunities for crop allele introgression in the field (e.g., [28], [49]). Also, while more competitive conditions appear to facilitate the introgression of crop alleles into wild populations, some cross types (e.g., the F_2_) may particularly benefit due to their unique characteristics. We encourage further research into the complexities of factors influencing introgression of novel alleles into wild populations across the landscape.

## Supporting Information

Figure S1 **Combined effects of density of wild sunflower, frequency of hybrid sunflower, and cross type on number of mature heads.**
(PDF)

Table S1 **Sample sizes for number of focal seeds that emerged (before slash) or that survived to reproduce (after the slash).**
(PDF)

Table S2 **ANOVA on number of viable seeds per head on crop-wild hybrid sunflowers grown in the field in Kansas, USA.**
(PDF)

Text S1 **Did our treatments alter the biotic environment?**
(PDF)

Text S2 **Estimates of seed per head and counts of number of heads per plant.**
(PDF)
