# Supplementary material for: Fitness of Crop-Wild Hybrid Sunflower under Competitive Conditions: Implications for Crop-to-Wild Introgression
Source: PLoS One. 2014 Oct 8;9(10):e109001. doi: 10.1371/journal.pone.0109001 (PMC4189920; doi:10.1371/journal.pone.0109001)

Supplementary Figure S1. Combined effects of density of wild sunflower, frequency of hybrid sunflower, and cross type on number of mature heads. Values are backtransformed least squares means with 95% confidence intervals (only upper portion of interval is shown). Values sharing the same letter are not significantly different based on a Tukey-Kramer multiple comparisons test. Methods: In addition to the overall model explained in the text, the analysis of numbers of heads (a per plant variable) included a factor to take into account the presence and source of apical meristem damage, which tended to enhance head production. Data was transformed to its natural logarithm to improve the distribution of the residuals.

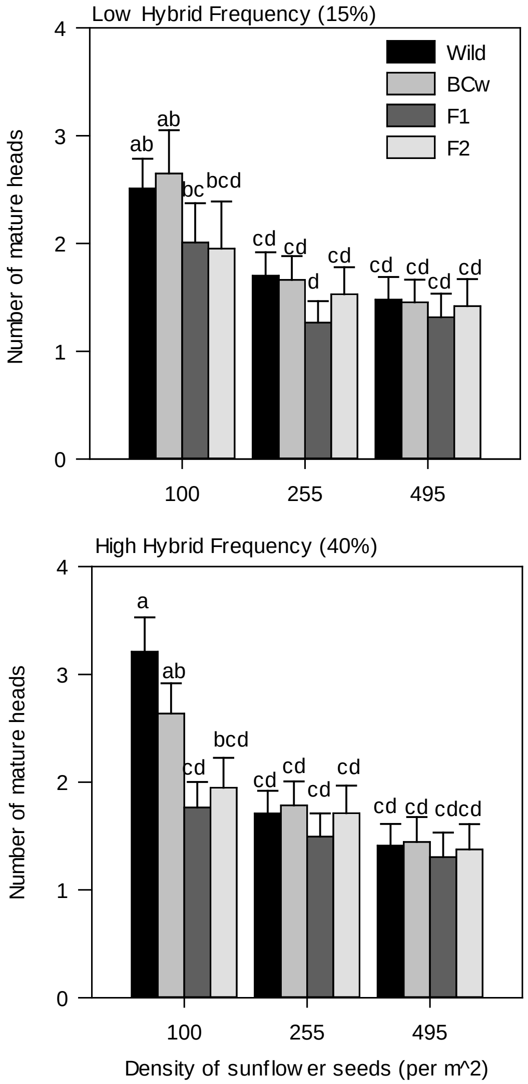

Supplement: Figure S1 — Combined effects of density of wild sunflower, frequency of hybrid sunflower, and cross type on number of mature heads. (PDF) [file pone.0109001.s001.pdf]
