# Supplementary material for: Fitness of Crop-Wild Hybrid Sunflower under Competitive Conditions: Implications for Crop-to-Wild Introgression
Source: PLoS One. 2014 Oct 8;9(10):e109001. doi: 10.1371/journal.pone.0109001 (PMC4189920; doi:10.1371/journal.pone.0109001)
Supplement: Table S1 — Sample sizes for number of focal seeds that emerged (before slash) or that survived to reproduce (after the slash). (PDF) [file pone.0109001.s002.pdf]

Supplementary Table S1. Sample sizes for number of focal seeds that emerged (before slash) or that survived to reproduce (after the slash). Yellow cells are calculated from a possible total of 47-48 focal seeds planted rather than 106-108 for clear cells. Overall sample size for focal seeds was 4824. Cross type: W (wild x wild), F1 hybrid (wild x crop), F2 hybrid (F1 x F1), and BCw hybrid (wild x F1) generations (with the maternal parent indicated first).

				Density of Intraspecific Competitors											
				Low (100 sd per m^2)				Medium (255 sd per m^2)				High (495 sd per m^2)			
				Cross type				Cross type				Cross type			
				W	BCw	F1	F2	W	BCw	F1	F2	W	BCw	F1	F2
Frequency of Intraspecific Competitors	Lower (15%)	Interspecific Competition	Without	72/68	26/26	26/23	15/13	68/58	68/58	58/53	45/36	75/55	68/52	55/40	48/37
			With	71/63	29/27	24/21	18/15	76/68	61/54	48/43	44/35	75/53	71/53	53/41	42/28
	Higher (40%)	Interspecific Competition	Without	74/69	71/67	61/57	42/36	72/66	68/63	53/46	43/36	76/55	59/40	54/40	42/33
			With	76/71	68/62	49/45	47/44	71/64	73/59	60/52	48/42	73/56	62/39	44/32	49/39
