# Supplementary material for: Fitness of Crop-Wild Hybrid Sunflower under Competitive Conditions: Implications for Crop-to-Wild Introgression
Source: PLoS One. 2014 Oct 8;9(10):e109001. doi: 10.1371/journal.pone.0109001 (PMC4189920; doi:10.1371/journal.pone.0109001)
Supplement: Table S2 — ANOVA on number of viable seeds per head on crop-wild hybrid sunflowers grown in the field in Kansas, USA. (PDF) [file pone.0109001.s003.pdf]

Supplemental Table S2. ANOVA on number of viable seeds per head on crop-wild hybrid sunflowers grown in the field in Kansas, USA.

		Primary HQ				Primary LQ				Secondary HQ				Secondary LQ		
Source	Ndf	ddf	F	P		ddf	F	P		ddf	F	P		ddf	F	P
Frequency of Hybrids	1	155	1.69	0.1985		55	0.89	0.3492		55	0.13	0.7159		55	0.29	0.5948
Interspec Competitors	1	55	10.19	**0.0023**		55	0.07	0.7888		55	3.91	0.0529		55	0.21	0.6465
Density of Seeding	2	55	25.46	**<.0001**		55	1.7	0.1913		55	8.74	**0.0005**		55	0.37	0.6922
Freq*Interspec	1	55	0.4	0.5291		55	0.47	0.4959		55	0	0.9587		55	0.23	0.631
Freq*Dens	2	55	0.13	0.8803		55	1.86	0.166		55	4.23	**0.0196**		55	0.25	0.7781
Interspec*Dens	2	55	0.31	0.7342		55	0.24	0.7856		55	0.25	0.7819		55	0.59	0.5574
Freq*Interspec*Dens	2	55	1.23	0.3006		55	0.44	0.6436		55	0.11	0.8949		55	0.82	0.4458
Cross Type	3	175	13.02	**<.0001**		108	14.63	**<.0001**		114	33.25	**<.0001**		84	8.59	**<.0001**
Freq*Cross	3	175	0.59	0.6216		108	0.4	0.7558		114	0.2	0.8941		84	0.11	0.9551
Interspec*Cross	3	175	0.24	0.8693		108	0.97	0.4078		114	0.53	0.6614		84	0.18	0.9081
Dens*Cross	6	175	1.17	0.3267		108	1.08	0.3765		114	0.59	0.7343		84	1.79	0.1112
Freq*Interspec*Cross	3	175	1.15	0.3309		108	4.88	**0.0032**		114	0.02	0.9963		84	0.22	0.8805
Freq*Dens*Cross	6	175	0.72	0.6318		108	0.34	0.9116		114	0.99	0.4377		84	1.42	0.2184
Interspec*Dens*Cross	6	175	0.47	0.8312		108	0.34	0.9129		114	0.86	0.5239		84	0.83	0.5471
Freq*Interspec*Dens*Cross	6	175	0.97	0.4439		108	0.92	0.4844		114	1.26	0.28		84	0.66	0.6784

Notes: Primary and secondary high (HQ) and low (LQ) quality head data analyzed separately. Only data from plants who survived to reproduce were employed in the ANOVA using Restricted Maximum Likelihood. Data include only primary and first secondary heads and were natural log transformed. P-values <0.05 are bolded as significant. nnf, numerator degrees of freedom; ddf, denominator degrees of freedom.
