# Supplementary material for: Fitness of Crop-Wild Hybrid Sunflower under Competitive Conditions: Implications for Crop-to-Wild Introgression
Source: PLoS One. 2014 Oct 8;9(10):e109001. doi: 10.1371/journal.pone.0109001 (PMC4189920; doi:10.1371/journal.pone.0109001)
Supplement: Text S1 — Did our treatments alter the biotic environment? (PDF) [file pone.0109001.s004.pdf]

Supplementary Text S1. Did our treatments alter the biotic environment?

In order to confirm that our experimental treatments had manipulated the biotic conditions as hoped, we quantified variation in plant density, interspecific competitors, and hybrid frequency. We took data at the plot level, rather than on each focal seedling.

First, to quantify plant density as a product of manipulating seed density, we counted stems of focal and non-focal sunflower plants found within two 10 cm x 10 cm quadrats in opposite corners of the plots. These counts were averaged within a plot for analysis. We successfully manipulated the density of sunflowers (ANOVA density effect on number of sunflower stems: $F_{2,55}=68.17, P<0.0001$). At low density (100 seeds/m_2), we averaged 7.5 sunflowers within 10 cm x 10 cm quadrats, 13.1 sunflowers at intermediate density (255 seeds/m_2), and 19.7 sunflowers at high density (495 seeds/m_2; each ± s.e. 0.74 and all significantly different with Tukey-Kramer adjustment.

Second, to quantify the presence of interspecific competitors (i.e., to see if our weeding treatment worked), we estimated percent cover of non-sunflower species from both sides of the plot and at three levels within the canopy (low, medium, and high); we present here the average of all estimates. By weeding out interspecific competitors, we altered the presence of non-sunflowers in the plots (ANOVA effect of interspecific competition on percent cover of non-sunflowers: $F_{1,55}=230.61, P<0.0001$). Weeded plots only had 1.8% cover (s.e. = 0.92%) by non-sunflowers late in the season (day 134), while weeded plots had 18.3% non-sunflower cover (s.e. = 0.92).

Finally, to observe the increased frequency of hybrids, we used the fact that hybrid plants flower earlier than wild ones and observed the number of flowering heads in the plots from the first day of flowering (day 117) until most flowering was complete (day 186). These flowering

data were collected only in the presence of vegetation in three of the six blocks. During the first flowering flush (days 117-134), which corresponds to the period of when F_1 and F_2 cross types tended to begin to flower (data not shown), there were significantly more flowers in the high hybrid frequency plots: high frequency, 24.1 flowers per plot vs. low frequency, 8.6 flowers per plot (backtransformed lsmeans) (day 127, ANOVA, log transformed data, effect of hybrid frequency: $F_{1,6}=20.79$, $P=0.0039$).
