# Supplementary material for: Fitness of Crop-Wild Hybrid Sunflower under Competitive Conditions: Implications for Crop-to-Wild Introgression
Source: PLoS One. 2014 Oct 8;9(10):e109001. doi: 10.1371/journal.pone.0109001 (PMC4189920; doi:10.1371/journal.pone.0109001)
Supplement: Text S2 — Estimates of seed per head and counts of number of heads per plant. (PDF) [file pone.0109001.s005.pdf]

Supplementary Text S2. Estimates of seed per head and counts of number of heads per plant

Estimates of seed production per plant depend on number of heads produced and the number of seeds per head. We were able to use direct counts of numbers of heads. However, we needed to estimate seeds per head, which was a two-step process. For all analyses, we used the natural log of seeds per head as a response variable to improve the distribution of the residuals.

Seeds per head: The first step was to understand which factors affected seeds per head. (Analyses were performed only on number of viable seeds; damaged seeds were ignored.) To do this, we used data from all focal plants that had produced at least one mature head. Overall, we found that number of viable seeds per head was most heavily influenced by whether a head was classified as high or low quality and whether it was a primary or secondary head. Low quality heads were common (25-50% of heads). However, our experimental treatments were also important, especially density of intraspecific competition, presence of interspecific competition, and cross type (Supplementary Table S1). (Frequency was also significant in interactions for some head types, but had a minimal effect, so we did not consider it further.)

The second step was to predict the seeds per head for low or high quality, primary or secondary heads depending on the combination of treatments in which it grew. For this, we used the backtransformed least squares means values. These back-transformed values of seeds per head were then plugged into the equation described in the text for estimating seeds per plant.
